# De-Intensification Strategies in Non-Muscle-Invasive Bladder Cancer: Outcomes and Cost Impact of In-Office Bladder Fulguration

**DOI:** 10.3390/jcm15051939

**Published:** 2026-03-04

**Authors:** Maria Teresa Melgarejo Segura, Miguel Herraez Marcos, Maria Carmen Cano Garcia, Alberto Zambudio Munuera, Patricia Rodriguez Parras, Miguel Angel Arrabal Polo

**Affiliations:** 1 Department of Urology, San Cecilio Clinical University Hospital of Granada, Av. Dr. Jesús Candel Fabregas, s/n, 18014 Granada, Spain; 2 ibs.GRANADA Biosanitary Research Institute, Av. de Madrid, 15, Beiro, 18012 Granada, Spain

**Keywords:** bladder cancer, non-muscle invasive bladder cancer, laser therapy, holmium laser, outpatient care, neoplasm recurrence

## Abstract

**Background/Objectives:** Non-muscle invasive bladder cancer (NMIBC) is characterized by high recurrence rates, requiring frequent diagnostic and therapeutic interventions. This study evaluates the feasibility, safety, oncological outcomes, and economic impact of implementing an in-office laser bladder tumor fulguration protocol. **Methods:** A descriptive, longitudinal study was conducted between 2020 and 2025 on 65 patients with recurrent NMIBC. Procedures were performed in an outpatient setting under local anesthesia using a flexible cystoscope and a Holmium:YAG (Ho:YAG) laser. The primary endpoint was recurrence-free survival. Secondary endpoints included complication rates (Clavien–Dindo) and a cost-analysis comparison with conventional transurethral resection of the bladder (TURBT). **Results:** The mean age was 69.4 years, with 89.2% of patients classified as ASA ≥ 2. After a median follow-up of 20.3 months, the recurrence rate was 33.8%, with 0% progression. Most procedures (95.4%) had no complications; only 4.6% presented Clavien–Dindo grade 1 events. Adjuvant mitomycin C was administered in 93.8% of cases. The cost analysis demonstrated substantial economic advantages, with costs reduced by 89.7% versus the 24 h admission model and 82.1% versus the day-surgery model according to regional health-system tariffs. **Conclusions:** In-office laser fulguration is a safe, effective, and economically sustainable alternative to traditional TURBT for selected low-risk recurrences. It optimizes hospital resources, minimizes anesthetic risk in comorbid patients, and maintains favorable oncological control.

## 1. Introduction

Bladder cancer is the ninth most common neoplasm worldwide, with more than half a million new cases diagnosed annually [1]. Approximately 75% of these cases are non-muscle invasive bladder cancers (NMIBC) [2]. The standard treatment for NMIBC across all risk groups is transurethral resection of the bladder (TURBT), which allows for complete lesion resection and accurate tumor staging [3].

The clinical challenge arises because, while NMIBCs have a lower risk of progression and cancer-specific mortality compared to muscle-invasive tumors, they are characterized by high recurrence rates [3]. Consequently, these patients have high survival rates which, coupled with increasing life expectancy, necessitates prolonged follow-up involving repeated diagnostic tests, such as cystoscopy, and multiple therapeutic interventions.

This burden results in a decline in quality of life [4], particularly in increasingly elderly patients with multiple comorbidities, and represents a significant financial strain on the healthcare system [5]. Within this framework, strategies for de-intensifying follow-up and treatment have emerged, including in-office fulguration of recurrent tumors [6].

This study presents the implementation of a bladder tumor fulguration protocol and evaluates the oncological outcomes, safety, and economic impact of introducing this technique at our center.

## 2. Materials and Methods

### 2.1. Study Design and Patient Selection

A descriptive study was conducted on patients who underwent bladder tumor fulguration at our center between 2020 and 2025. The primary objective was to determine the recurrence-free survival rate. Secondary objectives included the evaluation of procedure-related complications and the economic repercussions of implementing this technique at our center. Inclusion criteria were patients over 18 years of age with the capacity to provide informed consent, presenting with recurrent non-muscle invasive bladder tumors (NMIBC) of papillary morphology, with a diameter of less than 3 cm and fewer than 10 lesions. Patients with primary tumors and/or high-risk tumors were also included if they were unable to tolerate general anesthesia or if histological findings would not alter clinical management. Exclusion criteria included patients with a history of non-urothelial histology, carcinoma in situ (CIS), or tumors with a solid appearance. Upper urinary tract evaluation was not required for this study.

### 2.2. Variables and Follow-Up

The variables collected were age, sex, smoking status, anticoagulant/antiplatelet therapy, diabetes, American Society of Anesthesiologists (ASA) physical status, pathological stage, grade, primary or recurrent status, previous intravesical treatments, tumor size, number of tumors, and time to recurrence. Time to recurrence was defined as the period from fulguration until the detection of a new tumor during follow-up. Furthermore, subsequent intravesical treatments and complications within the first 30 days post-procedure were recorded, the latter categorized using the Clavien–Dindo classification, along with any emergency department visits.

For cost comparison, official prices established in the catalog of public prices for healthcare services provided in centers of the Andalusian Public Health System (SSPA) were used. These prices are published in the Official Gazette of the Regional Government of Andalusia (BOJA) and are available through the Andalusian Regional Government open data portal [7].

Statistical analyses were performed using IBM SPSS Statistics, version 26.0 (IBM Corp., Armonk, NY, USA). Continuous variables were expressed as mean ± standard deviation, and categorical variables as frequencies and percentages. Time-to-event analyses were conducted using Kaplan–Meier curves, with comparisons performed using the log-rank test. A *p*-value < 0.05 was considered statistically significant.

### 2.3. Procedure and Patient Preparation

After verifying informed consent and confirming recent coagulation study results, anticoagulant or antiplatelet therapies were managed according to the Spanish Society of Cardiology consensus [8].

All procedures were performed in an outpatient setting. Prophylactic intravenous ciprofloxacin (500 mg) was administered. No systemic analgesia or sedation was used. Following sterile field preparation, local anesthesia was applied intraurethrally using two doses of lidocaine gel (12.5 g each) five minutes apart. A bladder catheter was then inserted for complete drainage, followed by the intravesical instillation of 10 mL of 2% lidocaine (200 mg) diluted in 100 cc of normal saline, which was retained for 10 min.

### 2.4. Surgical Technique and Follow-Up

With the patient in the lithotomy position, a single-use flexible cystoscope (Ambu^®^ aScope™ 4 Cysto, 16.2 Fr, Ambu GmbH, Bad Nauheim, Germany) was introduced using normal saline as irrigation fluid. A systematic cystoscopy was performed to confirm the number, size, and location of the lesions; narrow-band imaging (NBI) was not routinely used. Biopsies of papillary recurrences were not systematically taken; when required, they were obtained prior to fulguration.

Fulguration was performed using a Holmium:YAG (Ho:YAG Dornier Medilas^®^ H Solvo^®^ 35 (Dornier MedTech GmbH, Argelsrieder Feld 7, D-82234 Wessling, Germany) laser with a 270-micron fiber (energy: 0.8 J; frequency: 12 Hz) in long-pulse mode (soft-tissue). The exophytic component was first ablated using “brush-stroke” movements. Subsequently, the base and the surrounding healthy tissue margin were coagulated, maintaining the fiber at a distance of approximately 2 mm to minimize the risk of tumor seeding.

Post-procedure, a single intravesical dose of 40 mg mitomycin C (diluted in 50 mL distilled water) was administered and retained for one hour with postural changes. Patients were discharged after voiding the chemotherapeutic agent at the clinic. Follow-up, including a control cystoscopy at three months, was conducted in accordance with the European Association of Urology (EAU) guidelines [3].

## 3. Results

### 3.1. Descriptive and Clinical Characteristics of the Cohort

A total of 65 patients undergoing in-office bladder fulguration were included. The mean age of the cohort was 69.4 years (SD: 11.68), with a median age of 70 years (range: 38–94), and a predominance of male patients (70.8%). Regarding baseline status, 89.2% of patients had an American Society of Anesthesiologists (ASA) physical status ≥ 2 (ASA 2: 44.6%; ASA 3: 41.5%; ASA 4: 3.1%). In terms of smoking status, 58.5% were former smokers, 13.8% current smokers, and 27.7% reported no history of tobacco use.

From an oncological perspective, most tumors were low grade (78.1%). The most frequent primary T stage was Ta (73.4%), followed by T1 (26.6%). Regarding tumor burden, 46.2% of patients had a single lesion, whereas 53.8% presented with multifocal disease, including 12.3% with five or more lesions.

In terms of safety, 95.4% of procedures were not associated with postoperative complications (Clavien–Dindo grade 0), with only three grade 1 events (4.6%) and no complications of grade ≥ 2. Adjuvant treatment with mitomycin C (MMC) was administered in 93.8% of patients (Table 1).

### 3.2. Oncological Outcomes: Recurrence and Progression

After a median follow-up of 20.3 months (range: 10.3–68.6), a recurrence rate of 33.8% (n = 22) was observed. No tumor progression events were documented during the follow-up period, resulting in a progression-free survival rate of 100%.

**Figure 1:**
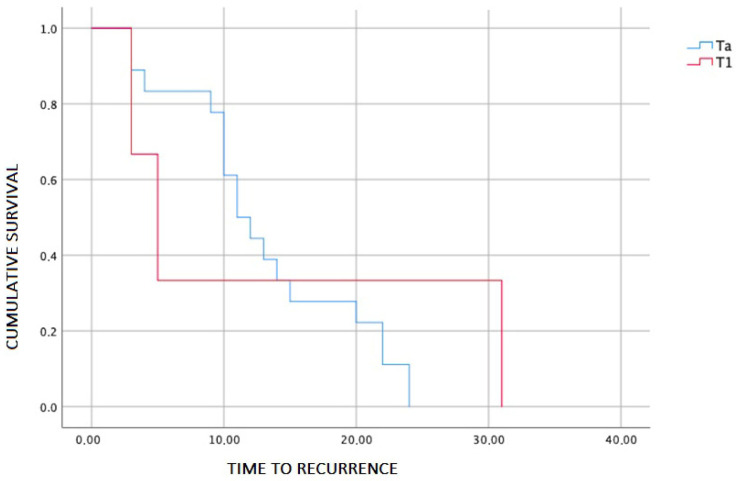
Recurrence-free survival according to primary T stage (Ta vs. T1). Kaplan–Meier curves depicting recurrence-free survival in patients with non–muscle-invasive bladder cancer treated with in-office bladder fulguration, stratified by primary T stage (Ta vs. T1).

In the bivariate analysis, no statistically significant associations were identified between tumor recurrence and the qualitative variables evaluated (sex, smoking status, tumor grade, primary T stage, and MMC administration; all *p* > 0.05). However, the primary T stage showed a clinically relevant trend, with a higher recurrence rate in Ta tumors (38%) compared with T1 tumors (17%) (Figure 1). This difference should be interpreted in the context of an unequal sample distribution, with a greater representation of Ta patients (n = 47) compared with T1 patients (n = 17).

Similarly, the size of the fulgurated lesion showed a trend toward larger values in the recurrence group, without reaching statistical significance (*p* = 0.061). The number of lesions did not differ significantly between patients with and without recurrence (*p* = 0.259).

### 3.3. Multivariate Analysis of Predictors of Recurrence

A binary logistic regression model was performed to identify independent predictors of tumor recurrence, including age, fulgurated tumor size, primary T stage (Ta vs. T1), and number of lesions. The overall model approached, but did not reach, statistical significance (χ^2^ = 9.10; *p* = 0.059), with a Nagelkerke R^2^ of 0.185.

None of the included variables reached individual statistical significance. Nevertheless, clinically relevant trends were observed. Age showed an inverse association with recurrence risk, with a progressive reduction in risk among older patients (OR 0.95; *p* = 0.067). Primary T stage demonstrated a higher risk of recurrence in Ta tumors compared with T1 tumors (OR 2.55; *p* = 0.201). Tumor size showed a positive but non-significant association (OR 1.10; *p* = 0.212), while the number of lesions was not independently associated with recurrence (*p* = 0.839).

In the multivariate analysis, age and primary T-stage were identified as the most clinically relevant variables for predicting recurrence. However, these associations did not reach statistical significance. The modest sample size limited the statistical power of the analysis, precluding the identification of these factors as independent predictors of recurrence within this cohort.

### 3.4. Cost Analysis and Institutional Impact

For cost calculation, the public prices established in the catalog of healthcare services of the Andalusian Public Health System (SSPA) in force during the study period were used. According to these tariffs, the cost of a conventional transurethral bladder procedure performed with 24 h hospital admission and without complications amounts to €2767.29. To address the increasing prevalence of day-case surgery, we also considered the SSPA tariff for transurethral bladder resection without overnight stay (Code II.2.5), which is valued at €1585.53.

Bladder fulgurations performed in the outpatient setting (in-office) were carried out without hospital admission, without the use of day-hospital beds, and without the need for a day-case surgery pathway, being strictly outpatient procedures with the patient attending and leaving the facility on their own. Although the SSPA catalog lacks a specific code for in-office laser fulguration, we estimated the per-procedure cost by combining the tariff for a urology consultant-led procedure (Code I.1.1.2: €17.84) and the basic nursing intervention (Code I.4.1: €15.93), totaling approximately €33.77, excluding depreciable laser fiber costs. Even when accounting for laser fiber consumption (approx. €200–250 per unit if not reused), the total cost per in-office procedure remains significantly lower (approx. €283.77) than both the 24 h admission and the day-surgery pathways.

This represents a cost reduction of 89.7% compared to the 24 h admission model and an 82.1% reduction compared to the day-surgery model. Consequently, the implementation of this strategy resulted in a relevant overall cost saving and optimized hospital resource utilization.

## 4. Discussion

Our findings support the safety and cost-effectiveness of a protocolized in-office bladder tumor fulguration program. This strategy maintains adequate oncological control while providing added institutional value through the systematic implementation of early intravesical chemotherapy and a formal health-system-based economic analysis compared with conventional surgical pathways.

The shift towards de-intensification in NMIBC is further supported by the emerging role of chemoablation. Recent evidence suggests that non-surgical ablative strategies, using mitomycin C or other agents, can effectively manage low-risk recurrences, reducing the need for repetitive instrumentation while maintaining oncological safety [9]. In this context, our results are consistent with previously published series [10]. Although no head-to-head studies exist comparing fulguration with other minimally invasive techniques, such as thermal ablation or active surveillance, it has been observed that, compared to traditional TURBT, patients report lower pain levels and improved quality of life [11]. In our protocol, we chose the Holmium laser due to its superior safety profile, characterized by a shallow tissue penetration of 0.5–1 mm [12].

A distinctive feature of our cohort is the high adherence to early postoperative mitomycin C (MMC) instillation. Contrary to literature trends, where single-dose MMC is underutilized despite being recommended for low-risk patients [10,13] and having a proven benefit in reducing recurrence [14], nearly all patients in our study completed this protocol. This general underutilization is often attributed to concerns regarding bladder perforation [15] or excessive bleeding; however, in our experience, fulguration minimizes perforation risk, allowing for safe administration. It is noteworthy that, while some authors associate MMC with an increase in urinary adverse events [16], no significant rates of irritative complications were observed in our cohort, reinforcing the tolerability of this regimen.

However, the success of de-intensification relies heavily on precise patient selection. The 2021 EAU risk stratification models have shown limitations in certain high-risk subgroups, emphasizing the need for individualized assessment rather than relying solely on traditional scoring systems [17]. To further refine this selection, the integration of urinary and molecular biomarkers represents the next frontier. Although not utilized in this cohort, molecular markers could provide a more objective basis for identifying patients suitable for in-office management versus those requiring radical intervention, potentially reducing the risk of understaging in the absence of systematic histology [18].

Beyond the findings of this study, fulguration offers significant intrinsic advantages. Economically, it is a highly efficient technique. Unlike conventional TURBT, it optimizes human resources by requiring only a urologist and a nurse, bypassing the need for an anesthesiologist. Additionally, even when compared to the more efficient day-surgery protocols—which still require operating theater time and specialized nursing—the in-office approach drastically reduces the economic burden by shifting the procedure to a standard consultation setting.

Furthermore, the learning curve is short, and the technique allows treatment of patients with anatomical comorbidities, such as urethral strictures.

From the patient’s perspective, the literature supports that laser fulguration facilitates an earlier return to daily activities [12]. By using local anesthesia, risks associated with general or regional anesthesia are eliminated—a critical factor for frail patients, as repeated surgical interventions increase mortality risk in this population [19]. This is particularly relevant given the complex interplay between systemic health and urothelial carcinogenesis; for instance, cardiovascular comorbidities have been linked to a higher incidence of bladder cancer, often defining a multimorbid patient profile [20] that benefits most from avoiding the physiological stress of conventional surgery. Ultimately, this approach optimizes the management of low-risk tumors, reducing both patient discomfort and healthcare costs [6]. Nevertheless, it is imperative to note that the technique has specific constraints: it is not recommended for tumors involving the ureteral orifice, high-grade tumors, or carcinoma in situ (CIS).

Despite its relevance, this study is not without limitations. First, the inclusion of selected high-risk tumors introduces some biological heterogeneity, which should be considered when generalizing the findings. Second, histology was not systematically obtained during fulguration, especially in recurrent T1 tumors, which may lead to underestimation of progression. Therefore, the reported 100% progression-free survival should be interpreted with caution. Third, the sample size is relatively small, which may restrict statistical power, and trends observed in multivariate analysis cannot be interpreted as predictive. Furthermore, the follow-up period might be considered insufficient for a disease with long-term recurrence patterns, limiting the ability to draw definitive conclusions about late recurrences. From a technical standpoint, the lack of systematic use of advanced imaging (Blue Light or NBI) represents a limitation, as these could improve the detection of suspicious areas. Additionally, the heterogeneity in adjuvant care (patients requiring further intravesical treatment due to clinical needs) acts as a confounding factor that could affect the analysis of pure oncological outcomes. Finally, there is a risk that the progression rate is underestimated since histological specimens are not routinely obtained during fulguration. Therefore, future prospective trials with larger cohorts and standardized imaging are warranted to further validate our findings.

## 5. Conclusions

The implementation of a laser fulguration protocol for bladder tumors in a tertiary center is a feasible and safe strategy that optimizes healthcare resources without compromising oncological outcomes. Due to its low economic impact, reduced anesthetic morbidity, and excellent patient tolerance, this technique is established as an efficient alternative for managing low-risk disease, allowing for immediate functional recovery.

## Figures and Tables

**Table 1:** Baseline characteristics of the study population.

**Variable**	**n (%) or Statistics**
Total number of patients	65
Age (years)	
Mean ± SD	69.4 ± 11.68
Median (range)	70 (38–94)
Sex	
Male	46 (70.8)
Female	19 (29.2)
Smoking status	
Former smokers	38 (58.5)
Never smokers	18 (27.7)
Current smokers	9 (13.8)
Tumor grade (n = 64)	
Low grade	50 (78.1)
High grade	14 (21.9)
Primary T stage (n = 64)	
Ta	47 (73.4)
T1	17 (26.6)
Complications (Clavien–Dindo)	
Grade 0	62 (95.4)
Grade 1	3 (4.6)
≥Grade 2	0 (0)
Mitomycin C administered	
Yes	61 (93.8)
No	4 (6.2)
Tumor recurrence	
No recurrence	43 (66.2)
Recurrence	22 (33.8)
Tumor progression	
Yes	0 (0)
No	65 (100)
Number of lesions	
1 lesion	30 (46.2)
2 lesions	17 (26.2)
3 lesions	6 (9.2)
4 lesions	4 (6.2)
≥5 lesions	8 (12.3)

## Data Availability

The data presented in this study are available on request from the corresponding author. The data are not publicly available due to privacy issues.

## Abbreviations

The following abbreviations are used in this manuscript:

ASA	American Society of Anesthesiologists
BOJA	Official Gazette of the Regional Government of Andalusia
CIS	Carcinoma in situ
EAU	European Association of Urology
Ho:YAG	Holmium-doped Yttrium Aluminum Garnet
MMC	Mitomycin C
NBI	Narrow-Band Imaging
NMIBC	Non-Muscle Invasive Bladder Cancer
OR	Odds Ratio
SSPA	Andalusian Public Health System
TURBT	Transurethral Resection of the Bladder
